# Two cases of acute respiratory failure following SARS‐CoV‐2 vaccination in post‐COVID‐19 pneumonia

**DOI:** 10.1002/rcr2.995

**Published:** 2022-07-04

**Authors:** Tomohiro Bando, Reoto Takei, Yoshikazu Mutoh, Hajime Sasano, Yasuhiko Yamano, Toshiki Yokoyama, Toshiaki Matsuda, Kensuke Kataoka, Tomoki Kimura, Yasuhiro Kondoh

**Affiliations:** ^1^ Department of Respiratory Medicine and Allergy Tosei General Hospital Seto Japan; ^2^ Department of Infectious Diseases Tosei General Hospital Seto Japan

**Keywords:** acute exacerbation of interstitial lung disease, COVID‐19, interstitial lung disease, vaccination

## Abstract

Severe acute respiratory syndrome coronavirus 2 (SARS‐CoV‐2) vaccination is a very effective method of preventing infection and is recommended for people having recovered from coronavirus disease 2019 (COVID‐19). In this novel case report, we describe two patients with post‐COVID‐19 pneumonia who experienced acute respiratory failure and new bilateral ground‐glass opacities several days after receiving SARS‐CoV‐2 vaccination. Both patients were treated with methylprednisolone pulse therapy and recovered from the disease successfully. Indeed, post‐COVID‐19 patients can gain benefits from the vaccine, but vaccination at the early stage of recovery from COVID‐19 might be a risk for certain populations. These cases highlight a potential association between vaccination, interstitial lung disease and worsening of post‐COVID‐19 pneumonia. Further investigation and research examining the relationship between the timing of SARS‐CoV‐2 vaccination and potential risks in post‐COVID‐19 patients is recommended.

## Graphical abstract

The two cases reported here highlight a potential association between vaccination, interstitial lung disease and worsening of post‐coronavirus disease 2019 (COVID‐19) pneumonia. Further investigation and research examining the relationship between the timing of severe acute respiratory syndrome coronavirus 2 vaccination and potential risks in post‐COVID‐19 patients is recommended.

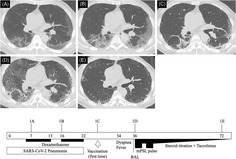

## INTRODUCTION

Coronavirus disease 2019 (COVID‐19) induced by severe acute respiratory syndrome coronavirus 2 (SARS‐CoV‐2) remains a top health concern even though some effective cures and vaccines have been developed. Several vaccines that have been developed and are currently under use can be administered even after COVID‐19. The Centers for Disease Control and Prevention (CDC) recommends SARS‐CoV‐2 vaccination for everyone older than 5 years of age, regardless of the history of SARS‐CoV‐2 infection.^1^ Although the optimal timing between infection and vaccination has not been fully elucidated, the CDC recommends waiting until after recovering from symptoms and ending isolation. We present two cases that are of interest for this discussion. Both patients had COVID‐19 pneumonia before receiving SARS‐CoV‐2 vaccination, and soon afterwards had new ground‐glass opacities (GGOs) and respiratory failure.

## CASE REPORT

### Patient 1

**FIGURE 1:**
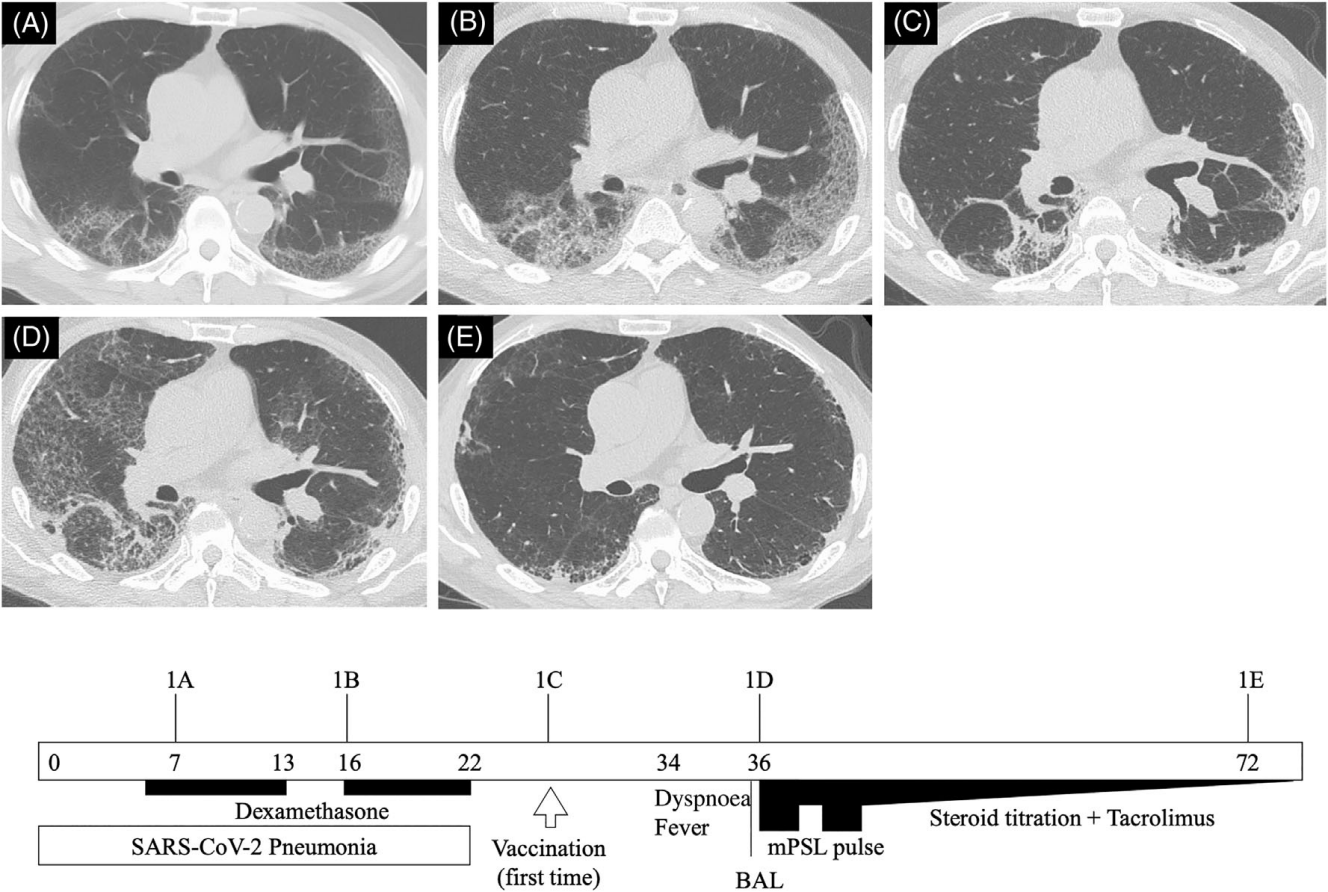
Simple clinical course of Case 1. Each HRCT corresponds to the figure numbers in the main text. Arabic numerals in the timeline square indicate the number of days since the onset of COVID‐19. BAL, bronchoalveolar lavage; COVID‐19, coronavirus disease 2019; HRCT, high‐resolution computed tomography; mPSL, methylprednisolone; SARS‐CoV‐2, severe acute respiratory syndrome coronavirus 2

The first patient was a 60‐year‐old male with a past medical history of hypertension and diabetes mellitus and a former smoking history of 46 pack‐years. He was diagnosed with SARS‐CoV‐2 pneumonia (Figure 1A) and treated with oral dexamethasone for 7 days. Then, because of continuing fatigue, he was treated with dexamethasone for another 7 days. He received his first BNT162b2 vaccine 26 days after the onset of SARS‐CoV‐2 pneumonia. Figure 1B,C shows his follow‐up high‐resolution computed tomography (HRCT), which indicates post‐COVID‐19 interstitial lung disease (ILD). Eight days after vaccination, he developed dyspnoea on exertion and a fever reaching 39.9°C. Ten days after vaccination, he was brought to the emergency department with peripheral oxygen saturation of 75% in ambient air. Physical examination revealed fine crackles in the bilateral lung bases but no signs of leg oedema or clubbed fingers. Arterial blood gas analysis in ambient air revealed hypoxaemia and respiratory alkalosis (pH 7.476, partial pressure of carbon dioxide [PaCO_2_] 28.6 Torr, partial pressure of oxygen [PaO_2_] 43.0 Torr, HCO_3_^−^ 21.1 mmol/L). Laboratory findings were white blood cells 8300 cells/μl, C‐reactive protein 20.12 mg/dl, lactate dehydrogenase 329 U/L, Krebs von den Lungen‐6 (KL‐6) 686 U/ml, surfactant protein‐D 354 ng/ml and ferritin 52.6 ng/ml. His chest HRCT showed diffuse bilateral GGOs superimposed on background due to post‐COVID‐19 pneumonia (Figure 1D).

SARS‐CoV‐2 polymerase chain reaction (PCR) testing was positive on admission, but the main aetiology did not seem to be SARS‐CoV‐2 since his threshold cycle (Ct value) for SARS‐CoV‐2 was over 35. Bronchoalveolar lavage (BAL) on the day after admission showed 18% neutrophils, 41% lymphocytes and 6.5% eosinophils. BAL fluid culture showed no evidence of bacterial infection.

His previous abdominal CT, taken 6 months earlier, showed bilateral subpleural reticulation in his lower lungs. This suggests pre‐existing fibrotic change or interstitial lung abnormalities without aetiology based on routine evaluations for ILDs. Moreover, HRCT before vaccination showed remaining subpleural consolidation and reticulation caused by COVID‐19, suggesting lung fibrosis (Figure 1C).

We diagnosed acute exacerbation (AE) of ILD (AE‐ILD) and treated him with a combination of corticosteroid (two cycles of methylprednisolone 1000 mg/day for 3 days followed by gradual tapering from 1 mg/kg/day of methylprednisolone to 10 mg/day of prednisolone for 30 days) and tacrolimus. On day 8 of admission, his respiratory condition had improved and he needed no oxygen at rest. The widespread GGO observed on his HRCT on admission was ameliorated with subpleural reticulation remaining in his lower lungs (Figure 1E).

### Patient 2

**FIGURE 2:**
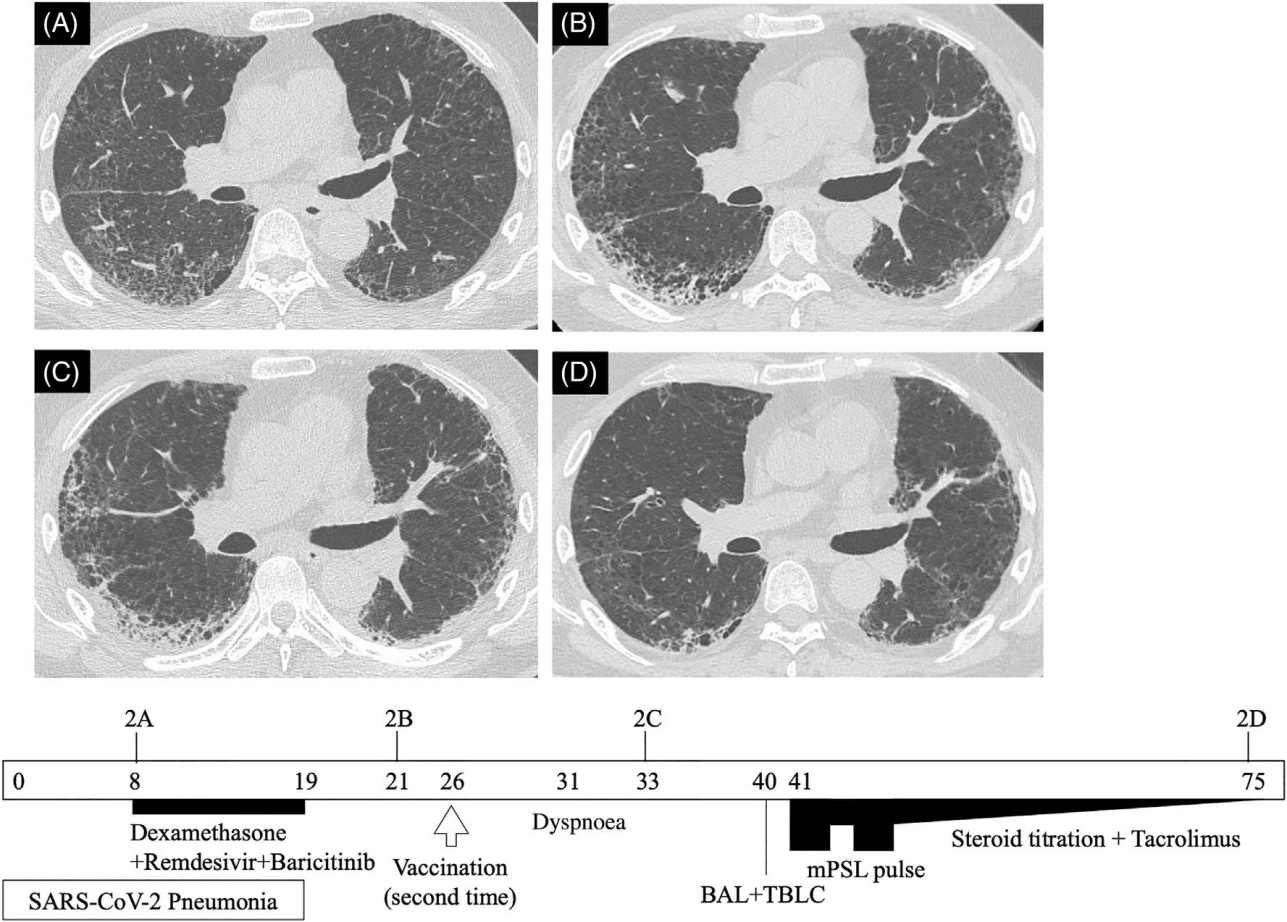
Simple clinical course of Case 2. Each HRCT corresponds to the figure numbers in the main text. Arabic numerals in the timeline square indicate the number of days since the onset of COVID‐19. BAL, bronchoalveolar lavage; COVID‐19, coronavirus disease 2019; HRCT, high‐resolution computed tomography; mPSL, methylprednisolone; SARS‐CoV‐2, severe acute respiratory syndrome coronavirus 2; TBLC, transbronchial lung cryobiopsy

The second case is of a 67‐year‐old man brought to the emergency department with shortness of breath and dyspnoea on exertion after receiving his second SARS‐CoV‐2 vaccination. He had a past medical history of rheumatoid arthritis (RA) treated with methotrexate, 5 mg of prednisolone and iguratimod by a local physician. Four days after his first BNT162b2 vaccination, he was diagnosed with SARS‐CoV‐2 pneumonia by PCR test (Figure 2A). He was admitted to our hospital 8 days after onset and treated with dexamethasone, remdesivir and baricitinib. At discharge, his HRCT showed bilateral subpleural reticulation and consolidation due to sequelae of COVID‐19 pneumonia (Figure 2B).

After discharge, there was mild exertional dyspnoea which did not affect his activities of daily living. He received his second SARS‐CoV‐2 vaccine on the 26th day from onset (30th day from the first vaccination). Five days after his second vaccination, he started to feel more shortness of breath and dyspnoea on exertion than usual, which led him to visit our hospital again. His vital signs were temperature of 36.5°C, respiratory rate of 20 breaths per minute and peripheral oxygen saturation of 92% in ambient air. Physical examination revealed bilateral fine crackles in the lung bases. Arterial blood gas analysis revealed hypoxaemia and respiratory alkalosis (pH 7.473, PaCO_2_ 35.0 Torr, PaO_2_ 62.0 Torr, HCO_3_^−^ 25.6 mmol/L). Laboratory findings were white blood cells 7500 cells/μl (neutrophils 78.0%, lymphocytes 16.0%, eosinophils 1.0%), C‐reactive protein 4.55 mg/dl, lactate dehydrogenase 260 U/L, KL‐6 812 U/ml and procalcitonin 0.088 ng/ml. His chest HRCT showed intensified reticulation and increased consolidation superimposed on reticulation and consolidation on background due to post‐COVID‐19 pneumonia (Figure 2C).

After admission, his respiratory condition and oxygen saturation gradually worsened and he needed supplemental oxygen. He tested positive for SARS‐CoV‐2 PCR, but the Ct value was over 35 for this patient as well. A bronchoscopy with BAL and transbronchial lung cryobiopsy (TBLC) were performed 7 days after admission for differential diagnosis. The results showed 16.5% neutrophils, 43.5% lymphocytes and 4.5% eosinophils. BAL fluid smear was negative for bacterial evidence. Histopathological findings from TBLC showed an organizing pneumonia (OP) pattern with mild infiltration of inflammatory cells, septal wall thickening and partial elastofibrosis.

The variety of possible diagnoses made a definite diagnosis rather difficult. Considering his progressing respiratory failure, insufficient treatment was to be avoided and high doses of corticosteroid and tacrolimus were initiated in line with the treatment for AE‐ILD (the same as in Case 1). This improved his respiratory condition and atrial blood gas analysis. His HRCT on discharge had also improved, but some reticulation and honeycomb structures remained (Figure 2D).

## DISCUSSION

Both the cases presented here showed aggravation of HRCT and respiratory failure some days after SARS‐CoV‐2 vaccination. To our knowledge, these are the first case reports describing acute respiratory failure and worsening of symptoms in post‐COVID‐19 patients receiving SARS‐CoV‐2 vaccination soon after recovery from COVID‐19.

Vaccination after infection with SARS‐CoV‐2 is reported to be effective in preventing reinfection.^2^, ^3^ The CDC, World Health Organization and national health institutes in other countries have approved vaccination in people with past COVID‐19 infection.^1^, ^4^ These statements do not indicate how long patients who have recovered from COVID‐19 should wait before receiving a vaccine, but the government of the United Kingdom recommends vaccination after clinical recovery from COVID‐19 (around 4 weeks after the onset of symptoms or diagnosis of COVID‐19).^4^ It has also been stated that persisting symptoms after COVID‐19 infection do not contraindicate vaccination, but recent symptoms and deterioration should be taken into account. Even though both of the presented patients still had some respiratory symptoms after treatment, such as mild dyspnoea on exertion and fatigue, we judged them to be sequelae of COVID‐19 and assessed the patients' conditions as stable. Therefore, we permitted them to receive the SARS‐CoV‐2 vaccination on the 26th day after the onset of COVID‐19.

However, the timing of vaccination could have been inappropriate. Both SARS‐CoV‐2 infection and vaccination are reported to induce immune reaction. Antibody titres are reported to peak within 3–5 weeks following infection^5^ and people who are symptomatic or hospitalized are reported to have higher antibody titres than others for at least several months.^6^, ^7^ These studies suggest that both our patients would have had higher antibody titre levels. It is quite possible that some sort of immune reaction related to COVID‐19 antibody and vaccine‐induced immunity was reactivated by the early vaccination.

Finally, the cases presented here have two common and noteworthy characteristics, which directly relate to differential diagnosis in these cases. One is the presence of post‐COVID‐19 pneumonia prior to vaccination and the other is the possibility of subclinical ILD before COVID‐19. The former suggests that vaccination may have triggered reactivation of inflammation in the presence of persistent inflammation during the recovery from COVID‐19.^8^ Myall et al. reported that some COVID‐19 patients experience persisting symptoms and OP pattern pneumonitis,^8^ and judging from the clinical course of the cases reported here, BAL results and pathological results (in the second case only), perhaps the major pathophysiology could have been recurrent OP rather than AE‐ILD. The latter implies the possibility of AE‐ILD, as we recently reported in a case study of AE of underlying fibrotic ILD.^9^ A similar case has been reported by Ghincea et al.^10^ The first patient here is suspected to have had pre‐existing ILD and in the second patient, a previous chest x‐ray showed reticulation in the lower lung fields, which raised the possibility of AE of RA associated with ILD. Post‐COVID‐19 ILD should also be considered when discussing this, although such cases sometimes improve over time and it is questionable whether these two cases had chronic fibrotic ILD at the moment of vaccination.^11^ Because vaccination in the early stage of recovery from COVID‐19 may induce an excessive immune response, careful consideration is needed for the timing of vaccination for people recovering from COVID‐19 pneumonia or those with underlying ILD before COVID‐19 infection.

In conclusion, these case reports highlight a potential association between SARS‐CoV‐2 vaccination and ILD, as well as the difficulty of deciding the timing for vaccination after COVID‐19 pneumonia. Further evaluation and research regarding the incidence and potential association with vaccination and ILD are recommended.

## AUTHOR CONTRIBUTION

All authors fulfil the criteria of authorship. Tomohiro Bando, Reoto Takei, Yoshikazu Mutoh and Yasuhiro Kondoh wrote the original manuscript. All authors contributed to revisions of the manuscript, provided final approval of the version to be published and agreed to be accountable for all aspects of the work.

## FUNDING INFORMATION

This study was partially supported by the Study Group on Diffuse Lung Disease, Scientific Research/Research on Intractable Diseases in the Ministry of Health, Labour and Welfare, Japan.

## CONFLICTS OF INTEREST

Tomohiro Bando, Reoto Takei, Yoshikazu Mutoh, Hajime Sasano, Yasuhiko Yamano, Toshiki Yokoyama, Toshiaki Matsuda, Kensuke Kataoka and Tomoki Kimura have nothing to disclose. Yasuhiro Kondoh reports funding from Nippon Boehringer Ingelheim Co. Ltd.; consulting fees from Asahi Kasei Pharma Corp., Shionogi & Co. Ltd., Boehringer Ingelheim Co. Ltd., Janssen Pharmaceutical K. K., Healios K. K. and Taiho Pharmaceutical Co. Ltd.; and payment for lectures from Asahi Kasei Pharma Corp., Shionogi & Co. Ltd., Boehringer Ingelheim Co. Ltd., Actelion Pharmaceuticals Ltd., DAIICHI SANKYO Co. Ltd., Bristol Myers Squibb, AstraZaneca K. K., Eisai inc., KYORIN Pharmaceutical Co. Ltd., Mitsubishi Tanabe Pharma and Novartis Pharma K. K., outside the submitted work.

## ETHICS STATEMENT

The authors declare that appropriate written informed consent was obtained for the publication of this manuscript and accompanying images.

## Data Availability

Data sharing is not applicable to this article as no new data were created or analysed in this study.

## References

[1] CDC : Frequently Asked Questions About COVID‐19 Vaccination. Available from: https://www.cdc.gov/coronavirus/2019-ncov/vaccines/faq.html. Cited: 12 March 2022.

[2] Hammerman A , Sergienko R , Friger M , Beckenstein T , Peretz A , Netzer D , et al. Effectiveness of the BNT162b2 vaccine after recovery from Covid‐19. N Engl J Med. 2022 Feb 16;386:1221–9. 10.1056/NEJMoa2119497 35172072 PMC8908846

[3] Cavanaugh AM , Spicer KB , Thoroughman D , Glick C , Winter K . Reduced risk of reinfection with SARS‐CoV‐2 after COVID‐19 vaccination – Kentucky, May‐June 2021. MMWR Morb Mortal Wkly Rep. 2021;70(32):1081–3. 34383732 10.15585/mmwr.mm7032e1 PMC8360277

[4] GOV. UK: COVID‐19: The Green Book, Chapter 14a. Available from: https://www.gov.uk/government/publications/covid-19-the-green-book-chapter-14a. Cited: 12 March 2022.

[5] Post N , Eddy D , Huntley C , van Schalkwyk MCI , Shrotri M , Leeman D , et al. Antibody response to SARS‐CoV‐2 infection in humans: a systematic review. PLoS One. 2020;15(12):e0244126. 33382764 10.1371/journal.pone.0244126 PMC7775097

[6] He Z , Ren L , Yang J , Guo L , Feng L , Ma C , et al. Seroprevalence and humoral immune durability of anti‐SARS‐CoV‐2 antibodies in Wuhan, China: a longitudinal, population‐level, cross‐sectional study. Lancet. 2021;397(10279):1075–84. 33743869 10.1016/S0140-6736(21)00238-5 PMC7972311

[7] Röltgen K , Powell AE , Wirz OF , Stevens BA , Hogan CA , Najeeb J , et al. Defining the features and duration of antibody responses to SARS‐CoV‐2 infection associated with disease severity and outcome. Sci Immunol. 2020;5(54):eabe0240. 33288645 10.1126/sciimmunol.abe0240 PMC7857392

[8] Myall KJ , Mukherjee B , Castanheira AM , Lam JL , Benedetti G , Mak SM , et al. Persistent post‐COVID‐19 interstitial lung disease. An observational study of corticosteroid treatment. Ann Am Thorac Soc. 2021;18(5):799–806. 33433263 10.1513/AnnalsATS.202008-1002OC PMC8086530

[9] Bando T , Takei R , Mutoh Y , Sasano H , Yamano Y , Yokoyama T , et al. Acute exacerbation of idiopathic pulmonary fibrosis after SARS‐CoV‐2 vaccination. Eur Respir J. 2022 Feb 10;59(3):2102806. 35144990 10.1183/13993003.02806-2021 PMC8832376

[10] Ghincea A , Ryu C , Herzog EL . An acute exacerbation of idiopathic pulmonary fibrosis after BNT162b2 mRNA COVID‐19 vaccination: a case report. Chest. 2022 Feb;161(2):e71–3. 35131075 10.1016/j.chest.2021.07.2160 PMC8814523

[11] Ambardar SR , Hightower SL , Huprikar NA , Chung KK , Singhal A , Collen JF . Post‐COVID‐19 pulmonary fibrosis: novel sequelae of the current pandemic. J Clin Med. 2021 Jun 1;10(11):2452. 34205928 10.3390/jcm10112452 PMC8199255

